# Glutaminase-1 (GLS1) inhibition limits metastatic progression in osteosarcoma

**DOI:** 10.1186/s40170-020-0209-8

**Published:** 2020-03-05

**Authors:** L. Ren, V. Ruiz-Rodado, T. Dowdy, S. Huang, S. H. Issaq, J. Beck, H. Wang, C. Tran Hoang, A. Lita, M. Larion, A. K. LeBlanc

**Affiliations:** 1 Comparative Oncology Program, Center for Cancer Research, National Cancer Institute, National Institutes of Health, Bethesda, MD 20892 USA; 2 Metabolomics Section, NeuroOncology Branch, Center for Cancer Research, National Cancer Institute, National Institutes of Health, Bethesda, MD 20892 USA; 3 Pediatric Oncology Branch, Center for Cancer Research, National Cancer Institute, National Institutes of Health, Bethesda, MD 20892 USA; 4 Laboratory of Human Carcinogenesis, Center for Cancer Research, National Cancer Institute, National Institutes of Health, Bethesda, MD 20892 USA

**Keywords:** Osteosarcoma, Metastasis, Metabolism, Glutaminase, Metformin

## Abstract

**Background:**

Osteosarcoma (OS) is a malignant bone tumor that often develops during the period of rapid growth associated with adolescence. Despite successful primary tumor control accompanied by adjuvant chemotherapy, death from pulmonary metastases occurs in approximately 30% of patients within 5 years. As overall survival in patients remains unchanged over the last 30 years, urgent needs for novel therapeutic strategies exist. Cancer metastasis is characterized by complex molecular events which result from alterations in gene and protein expression/function. Recent studies suggest that metabolic adaptations, or “metabolic reprogramming,” may similarly contribute to cancer metastasis. The goal of this study was to specifically interrogate the metabolic vulnerabilities of highly metastatic OS cell lines in a series of in vitro and in vivo experiments, in order to identify a tractable metabolically targeted therapeutic strategy for patients.

**Methods:**

Nutrient deprivation and drug treatment experiments were performed in MG63.3, 143B, and K7M2 OS cell lines to identify the impact of glutaminase-1 (GLS1) inhibition and metformin treatment on cell proliferation. We functionally validated the impact of drug treatment with extracellular flux analysis, nuclear magnetic resonance (NMR) spectroscopy, and mass spectrometry. ^13^C-glucose and ^13^C-glutamine tracing was employed to identify specific contributions of these nutrients to the global metabolic profiles generated with GLS1 inhibition and metformin treatment in vivo.

**Results:**

Highly metastatic OS cell lines require glutamine for proliferation, and exposure to CB-839, in combination with metformin, induces both primary tumor growth inhibition and a distinct reduction in metastatic outgrowth in vivo. Further, combination-treated OS cells showed a reduction in cellular mitochondrial respiration, while NMR confirmed the pharmacodynamic effects of glutaminase inhibition in tumor tissues. We observed global decreases in glycolysis and tricarboxylic acid (TCA) cycle functionality, alongside an increase in fatty acid oxidation and pyrimidine catabolism.

**Conclusions:**

This data suggests combination-treated cells cannot compensate for metformin-induced electron transport chain inhibition by upregulating glutaminolysis to generate TCA cycle intermediates required for cell proliferation, translating into significant reductions in tumor growth and metastatic progression. This therapeutic approach could be considered for future clinical development for OS patients presenting with or at high risk of developing metastasis.

## Background

Cancer metastasis is estimated to be responsible for ~ 90% of cancer-associated deaths [1]. Increasingly, efforts are being directed at developing therapeutic strategies designed to limit or prevent metastasis [2]. Metastatic cancer cells must overcome significant hurdles to successfully colonize secondary sites, resulting in significant inefficiencies in this multistep biological process [3, 4]. Metabolic plasticity may allow metastatic cancer cells to cope with various stressful microenvironmental situations, which are believed to be distinct from those faced by cells during primary tumor formation; as such there is increasing evidence that altered metabolism is a driver of cancer metastasis [5–7]. Osteosarcoma (OS) is a highly aggressive malignancy of bone which affects ~ 900 pediatric and adolescent patients per year in the USA, with the primary cause of death being metastatic progression [5, 8] (https://www.cancer.org/cancer/osteosarcoma/about/key-statistics.html). We have previously shown that the expression of several distinct cellular metabolism genes was altered when highly metastatic OS cells lost their metastatic potential through inhibition of Ezrin phosphorylation [9]. We then hypothesized that highly metastatic cells may have a greater flexibility in managing energetic needs during times of stress and that metastatic success may be linked to cells’ ability to efficiently access and utilize energy. This led us to explore the potential for nutrient targeting as an anti-metastatic approach with potential translational value for OS patients.

Glutamine, the most abundant plasma amino acid, has many cellular fates and is a key nutrient that fuels the growth of many cancers primarily through its contribution to formation of tricarboxylic acid (TCA) cycle intermediates [10], which proliferating cells use for production of proteins and nucleotides [11, 12]. A critical step in the utilization of glutamine is its conversion to glutamate by the mitochondrial enzyme glutaminase (GLS). GLS is broadly expressed in a number of normal tissues and also thought to play a main role in cancer progression [13–15]. Glutamate and glutamate-derived metabolites support a number of crucial cellular pathways including the TCA cycle, redox balance, ATP generation, and amino acid synthesis [6, 12, 16]. Targeting glutamine utilization as an anti-cancer strategy shows promise for those tumor types which exhibit “glutamine addiction,” often associated with specific genomic alterations such as c-myc upregulation, KRAS mutations, mTOR upregulation, and NRF2 activation, as well as loss of tumor suppressors such as PTEN and RB1 [10]. CB-839 is a selective GLS1 inhibitor with antitumor activity across a variety of tumor types [14] and is the subject of many ongoing clinical trials. However, there is sparse literature describing the effect of CB-839 in sarcomas [17].

Metformin (1,1-dimethylbiguanide) is a widely used anti-diabetes drug that may have value as a repurposed drug for cancer therapy [18–20]. Recent evidence has suggested mitochondrial complex 1 inhibition is a central mechanism responsible for metformin’s inhibitory effects on cancer progression [21] and that complex I inhibition further impacts nicotinamide adenine dinucleotide (NAD^+^/NADH) homeostasis and aspartate levels, which are key components of orderly cell metabolism, proliferation, and maintenance of redox balance [22, 23]. Based on these findings, and the longstanding clinical experience with metformin in human diabetes patients, metformin is being evaluated, both alone and in combination strategies, in over 200 clinical studies in a variety of tumor types (www.clinicaltrials.gov).

## Methods

### Cell culture

Murine K7M2 [24], human 143B (ATCC, Mabassas, VA), and MG63.3 [25] OS cells were grown at 37 °C in 5% CO_2_ in DMEM supplemented with 10% fetal bovine serum and 2 mM glutamine. For nutrient deprivation experiments, cells were cultured in glucose-, glutamine-, and sodium pyruvate-free DMEM supplemented with varied concentrations of glucose, glutamine, and pyruvate.

### Cell proliferation assays

Cell proliferation assays were performed using the IncuCyte ZOOM system (Essen BioScience Inc). For the nutrient deprivation assay, 500 cells were seeded in each well of a 96-well plate and cultured with media containing varying concentrations of glucose, glutamine, and pyruvate for up to 6 days. Serial phase-contrast images were gathered and processed as the percentage of confluency to measure cell proliferation. Each data point represents the mean reading from sextuplicate analyses. All assays were conducted in duplicate. For drug treatment, the drug concentrations are indicated within the figures and text. Dimethyl sulfoxide (DMSO) was used as the vehicle for group comparisons. Statistical analysis was performed with GraphPad Prism.

### Sulforhodamine B (SRB) cell proliferation assay

Cells were plated in a 96-well plate at a density of 2500 cells per well in 100 μl of complete media and incubated overnight. Cells were then treated with glutaminase inhibitors CB-839, compound 968 and bis-2-(5-phenylacetamido-1,3,4-thiadiazol-2-yl) ethyl sulfide (BPTES; Sigma-Aldrich) at concentrations as indicated and processed after 48 h as previously described [26].

### Synergy Combination Index (CI) assay

SRB assay technique was employed for the Synergy Combination Index (CI) assay. Twenty-five combinations of CB-839 and metformin were tested. The data analysis was performed employing the method previously described in [27].

### Measurement of oxygen consumption rate

Oxygen consumption rates (OCR) were measured using a Seahorse Bioscience XF^e^96 Extracellular Flux Analyzer. MG63.3 cells were plated at 10,000 cells/well in XF96 cell culture microplates. Following attachment, cells were treated with (1) vehicle, (2) 1 μM CB-839 (Selleck Chemicals), (3) 5 mM metformin (Sigma-Aldrich), or (4) 1 μM CB-839 + 5 mM metformin and incubated for 24 h at 37 °C. Just prior to the Seahorse assay, growth media was replaced with 180 μL of Seahorse XF Base Media (Agilent) supplemented with glucose, glutamine, and sodium pyruvate, and the plate incubated in a 37 °C incubator lacking CO_2_ for 45 to 60 minutes. OCR was determined by performing the Cell Mito Stress Test (Agilent) according to the manufacturer’s specifications, as previously described [26].

### In vivo orthotopic xenograft tumor growth and spontaneous metastasis

All animal work was conducted with the approval of the Animal Care and Use Committee of the National Cancer Institute. Primary tumor growth was evaluated by orthotopic injection of 10^6^ MG63.3 human osteosarcoma cells/ 0.1 ml of Hank’s balanced salt solution (HBSS) into a parosseous site deep in the left caudal gastrocnemius of 6-week-old female SCID-Beige mice (Fox Chase CB17.B6-*Prkdc*^*scid*^*Lyst* ^*bg*^/Crl) as described previously [26]. For the spontaneous metastasis experiment, the treatment started approximately 1 month after tumor cell injection (primary tumors measuring 12–13 mm in diameter). Mice received gavage of vehicle (HPBCD, 2-hydroxypropyl-beta-cyclodextrin given twice daily), CB-839 (Calithera Biosciences) (200 mg/kg, twice daily), metformin (300 mg/kg, once a day), or combination of CB-839 and metformin, 7 days a week continuously. Drug doses were derived from the manufacturer’s data (for CB-839) and from prior literature [14]. The volume of orthotopic tumor growth was measured twice a week with digital calipers to obtain two diameters of the tumor sphere determined using the equation (*D* × *d*^2^)/6 × 3.12 (where *D* = the maximum diameter and d = the minimum diameter). Mouse body weights were measured weekly. When primary tumors of vehicle-treated mice reached 15–17 mm in diameter, experiments were terminated. Complete necropsy allowed confirmation of pulmonary metastases in all mice. Micro-metastases of lungs were examined and imaged with fluorescent inverted microscopy (Leica DMIRB). The areas of fluorescent lung metastases were calculated with ImageJ software. Statistical analysis was performed with GraphPad Prism.

### In vivo experimental metastasis

As previously described [28], tumor cells were harvested and prepared in HBSS. For tail vein injection assays, 10^6^ K7M2 cells or 10^4^ MG63.3 cells were intravenously injected into 5–6-week-old female BALB/c or SCID-Beige mice. Mice were either treated at Day 2 after tumor cell injection (early treatment) or at Day 9, when the micro-metastases were established in the lungs (late treatment). Mice were randomly divided into four cohorts (the experiments were repeated 2–3 times with *n* = 3–9), receiving daily gavage of vehicle (HPBCD, twice/day), CB-839 (200 mg/kg, twice/day), metformin (300 mg/kg, once/day), or combination of CB-839 and metformin. The experiments were terminated after 30 consecutive days of treatment. Lungs of treated mice were inflated and formalin-fixed. The whole lung fluorescent images were acquired via fluorescent stereomicroscopy (Leica MGFLIII). The percent of the lung occupied by metastases area/total lung area was calculated with ImageJ software. Lung metastases were also examined using H&E stained paraffin-embedded sections. Statistical analysis was performed with GraphPad Prism.

### ^13^C tracer studies of metabolism in xenograft tumors

For the ^13^C_6_-glucose tracer study, MG63.3 cells (10^6^/mouse) were orthotopically injected in SCID-Beige mice. Thirty days after injection, the mice were randomly divided into four cohorts (*n* = 3), receiving daily gavage of vehicle (HPBCD, twice/day), CB-839 (200 mg/kg, twice/day), metformin (300 mg/kg, once/day), or combination of CB-839 and metformin for 10 days. D-Glucose-^13^C_6_ (Cambridge Isotope Laboratories, Inc.) (25%) was prepared (20 mg) in 80 μl sterile PBS and injected through the tail vein into mice at 15 min intervals for 3 times (total = 332 μmol). Mice were euthanized 15 min after the last injection (45 min from the first injection). Tumors were removed, measured, and flash-frozen in liquid nitrogen. The same procedure was used for the ^13^C_5,_^15^N_2_-Glutamine (Sigma-Aldrich) tracer study. ^13^C_5,_^15^N_2_-Glutamine was prepared as a 36.2 mg/ml stock solution in sterile PBS and injected (200 μl, 7.24 mg) at 15 min intervals for 3 times (total = 142 μmol).

### Sample preparation for ^1^H-NMR

Frozen tumor samples were weighed and transferred to a glass vial for homogenization using a Polytron bench top homogenizer (Kinematica, Inc., Bohemia, NY) in a 1:2:2 water:methanol:chloroform solution. Identical solvent proportions were employed for metabolite extraction of cultured cells, although cell lysing was performed by 3 cycles of freeze-thawing, performing the latter in an ice-water sonication bath. After obtaining the first lysate in water only, 20 μL were put aside in order to analyze the protein content for further normalization. Samples were centrifuged at 12,000 rpm for 20 min. at 4 °C . The two resulting phases (upper aqueous polar and lower organic lipid) were separated and the protein interface was discarded. For NMR, the top (hydrophilic) layer was then transferred to a vial and dried under a stream of N_2_. The sediment was reconstituted in 180 μL of pH 7 phosphate buffer (75 mM) in 99.9% D_2_O containing TSP and 1% NaN_3_, spun-down at 10,000 rpm for 10 min. at 4 °C and the clear supernatant was then transferred to a 3-mm NMR tube. The bottom layer was dried as described above, but the dried sediment was resuspended in 180 μL of a 2:1 solution of CDCl_3_:CD_3_OD containing TMS.

### NMR spectral acquisition and processing

All spectra were acquired on a Bruker Avance III 600 MHz spectrometer (Structural Biophysics Laboratory, NCI, Frederick, Maryland, USA) operating at a probe temperature of 298 K. Single-pulse ^1^H NMR experiments were performed using the noesygppr1d (TopSpin 3.5, Bruker Biospin) pulse sequence for water suppression. For each spectrum, 128 scans were acquired, with a relaxation delay of 3 s, a spectral width of 10.8 KHz, and a time domain of 32 K points. Spectra were referenced to the TSP internal standard signal (s, δ = 0.00 ppm), zero-filled to 64 K points, and phased and baseline-corrected using ACD Labs Spectrus Processor 2016, and an exponential line broadening function of 0.30 Hz was applied. For quantification, ^1^H NMR resonance signals were normalized to the TSP singlet located at 0.00 ppm and to the tissue weight or protein content. 1D-HSQC spectra were acquired for 768 scans, a time domain of 3,5 K, a delay of 1.75 s, and a spectral width of 8 KHz. The spectral processing involved the application of exponential line broadening function of 4 Hz and a Gaussian function of 7.5 Hz.

For quantification of ^13^C-derived metabolites via NMR, 1D-HSQC spectra were acquired for 768 scans, a time domain of 3,5 K, a delay of 1.75 s, and a spectral width of 8 KHz. The spectral processing involved the application of exponential line broadening function of 4 Hz and a Gaussian function of 7.5 Hz. Areas under the curves displayed on the spectra were computed using the peak fitting option from ACD Labs Spectrus Processor 2016. Concentrations values arising from these procedures were analyzed using MetaboAnalyst 3.0 and R statistical software. A detailed description of all the methods and analysis involving LC-MS experiments can be found in Additional file 3 (Supplementary Methods).

## Results

### Proliferation of OS cells relies on glutamine

**Fig. 1:**
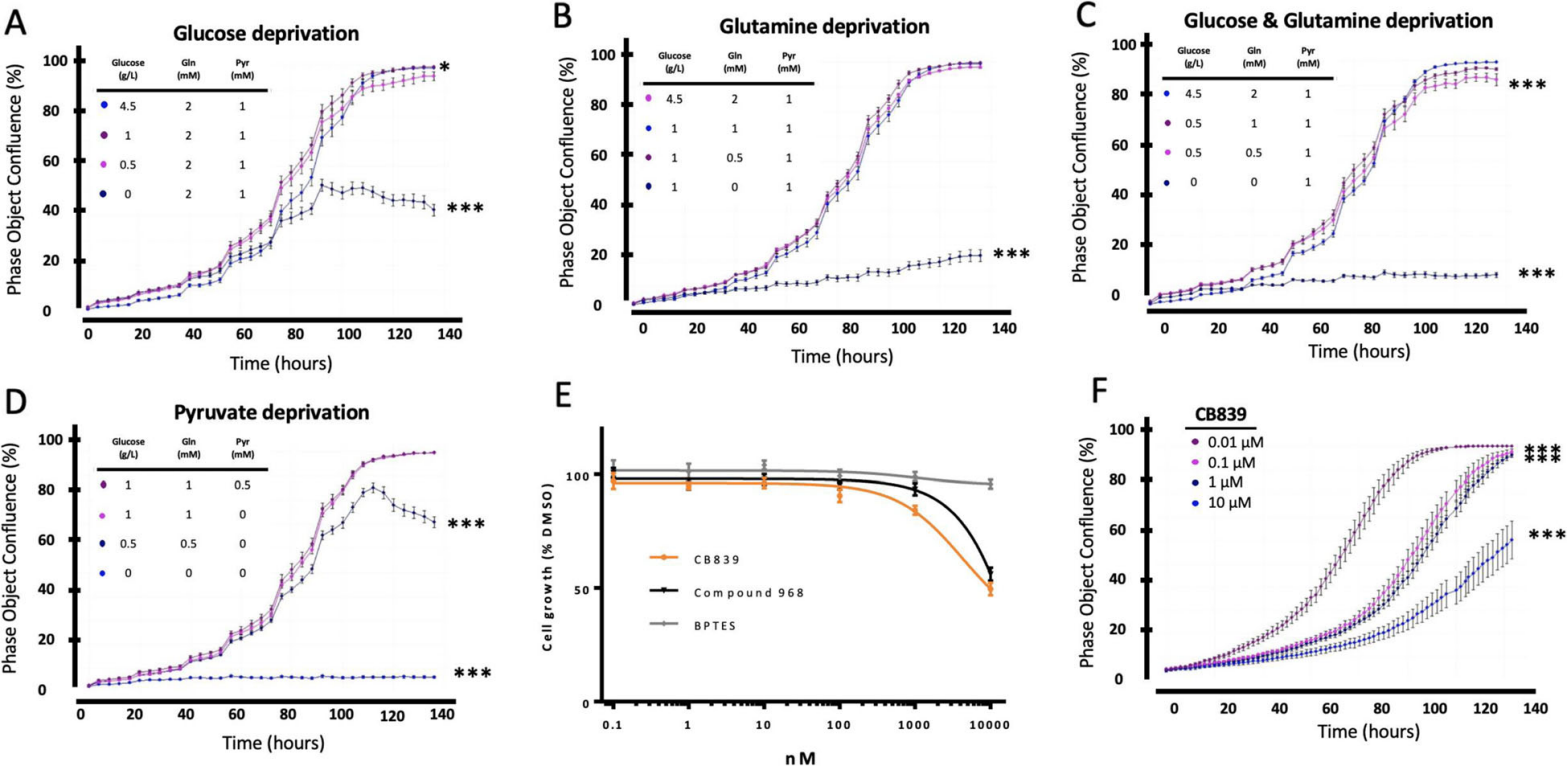
OS cells are sensitive to glutamine depletion. The proliferation responses of OS MG63.3 cells to the deprivation of three carbon source nutrients: glucose (**a**), glutamine (**b**), both glucose and glutamine (**c**), or pyruvate (**d**). **e** The growth inhibition of MG63.3 cells with the treatment of glutaminase inhibitors (Compound 968, CB-839 and BPTES) for 48 h is represented as percentage of DMSO-treated control. **f** The proliferation responses of OS MG63.3 cells to varies concentration of CB-839 treatment for 6 days. Statistic analysis used ANOVA followed by multiple comparisons “vehicle vs each treatment,” *p < 0.05, **p < 0.01, ***p < 0.001

To examine the dependency of OS cells on different carbon sources for growth, we first performed nutrient deprivation assays to analyze cellular proliferation in both murine (K7M2) and human (MG63.3) highly metastatic OS cell lines. As shown in Fig. 1d and Additional file 1: Figure S1, OS cell growth was not significantly affected when completely deprived of pyruvate from their culture medium as long as either glucose or glutamine were available. The complete elimination of glucose in the culture medium reduced cell growth significantly (Fig. 1a). Notably, OS cells were highly sensitive to glutamine deprivation, as negligible cell growth occurred in this context (Fig. 1b). When both glucose and glutamine were withdrawn from culture medium, the cell growth was completely inhibited (Fig. 1c). Similar results were seen in the murine OS cell line K7M2 (Additional file 1: Fig. S1).

A critical step in the utilization of glutamine is its conversion to glutamate by the mitochondrial enzyme glutaminase (GLS). We assessed response of MG63.3 cells to glutaminase inhibitors CB-839, compound 968, and BPTES (Fig. 1e). In a 48-h SRB cell proliferation assay, only a slight reduction in cell growth was observed up to 1 μM of CB-839 which is the pharmacologically achievable concentration in vivo [14], although concentrations above 1 μM for both compound 968 and CB-839 delayed the growth above 50%. Growth inhibition of MG63.3 cells was negligible upon BPTES treatment within this 48-h assay. With an extended period of treatment, a sustained inhibition of cell growth (> 50% growth inhibition in 6 days) was achieved with ≥ 10 μM CB-839 (Fig. 1f). Similar results were obtained with other OS cell lines (K7M2, HOS-MNNG, data not shown). This data demonstrates that although OS cells rely on glutamine for proliferation, use of a glutaminase inhibitor alone is not sufficient to inhibit OS cell growth in standard cell culture conditions. This is likely due to the cells’ ability to use alternate substrates to generate TCA cycle intermediates that are required for proliferation in vitro under standard growth conditions.

### Glutaminase inhibitor CB-839 in combination with metformin inhibits cell growth

Given that treatment with a glutaminase inhibitor alone was not sufficient to inhibit OS cell proliferation despite a dependence on glutamine, we examined whether a rational drug combination targeting cellular metabolism would be more effective. Metformin is a widely prescribed drug that can directly impact tumor cell metabolism, primarily by targeting mitochondrial complex I [21]. Recent reports of metformin combined with a glutaminase inhibitor suggested a synergistic effect in pancreatic and prostate cancers [20, 29]. Given these findings along with our previous work demonstrating that metformin significantly affects OS mitochondrial metabolism [30], we chose to evaluate the combination of metformin with CB-839 in the context of in vivo tumor formation (both primary tumor growth and metastatic progression).

**Fig. 2:**
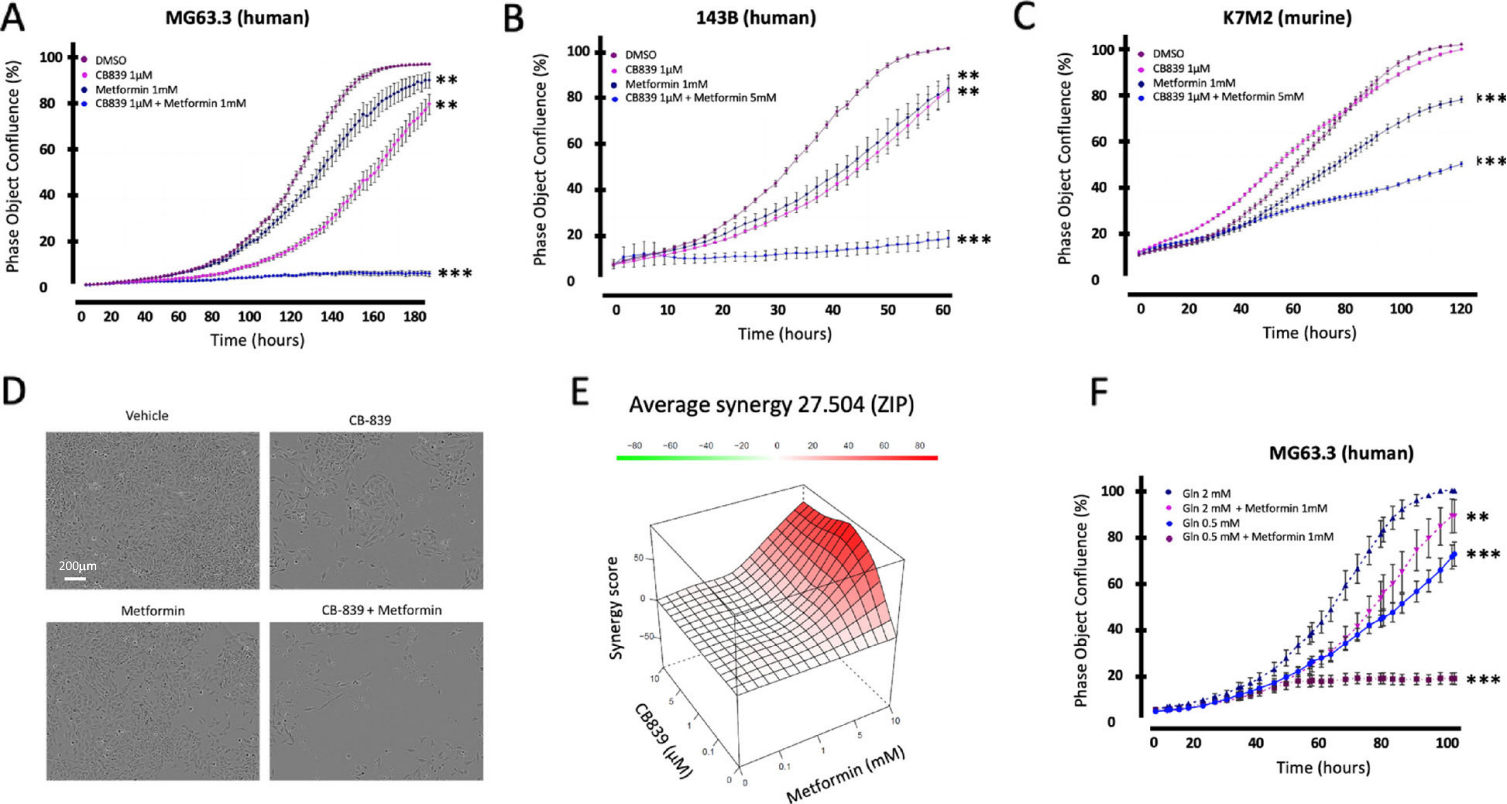
Inhibition of GLS with CB-839 or depletion of cellular glutamine in combination with metformin reduces OS cell growth. **a**–**d** The proliferation responses of OS cells to the treatments of CB-839 and metformin alone or in combination. OS cells of human MG63.3 (**a**), 143B (**b**), and murine K7M2 (**c**) were treated with vehicle, CB-839 (1 μM), metformin (1 mM for MG63.3 and 5 mM for 143B and K7M2) as a single agent or in combination for 3–7 days until the vehicle-treated cells reached 100% confluency. **d** Phase-contrast images of CB-839 and metformin treated MG63.3 cells. **e** Synergy combination index data. **f** The proliferation responses of MG63.3 cells to the deprivation of glutamine (0.5 mM) or in the combination with metformin (1 mM). Statistic analysis is used ANOVA followed by multiple comparisons “vehicle vs each treatment,” *p < 0.05, **p < 0.01, ***p < 0.001

Highly metastatic human MG63.3 (Fig. 2a) and 143B OS cell lines (Fig. 2b) along with murine K7M2 (Fig. 2c) OS cells were treated with the combination of metformin (1 or 5 mM) + CB-839 (1 μM) or each single agent. All cell lines tested exhibited a significantly greater growth inhibition with the combination therapy compared to monotherapy with either CB-839 or metformin (Fig. 2a–c). As shown in Fig. 2d the growth of MG63.3 cells was almost completely inhibited with 1 mM metformin +1 μM CB-839. A synergy combination index experiment also confirmed a synergistic effect exists with the combination of CB-839 and metformin (Fig. 2e). To further confirm the combination therapy effect, we tested if adding metformin to glutamine-deprived culture medium could enhance growth inhibition of OS cells. As shown in Fig. 2f, metformin (1 mM) did not change MG63.3 cell growth rate when added to standard culture medium (2 mM glutamine). However, a substantial reduction in cell growth occurred when adding metformin (1 mM) to glutamine-deprived medium (0.5 mM glutamine).

### Metabolic assessment of metformin, CB-839, and combination treatment on OS cells in vitro

**Fig 3:**
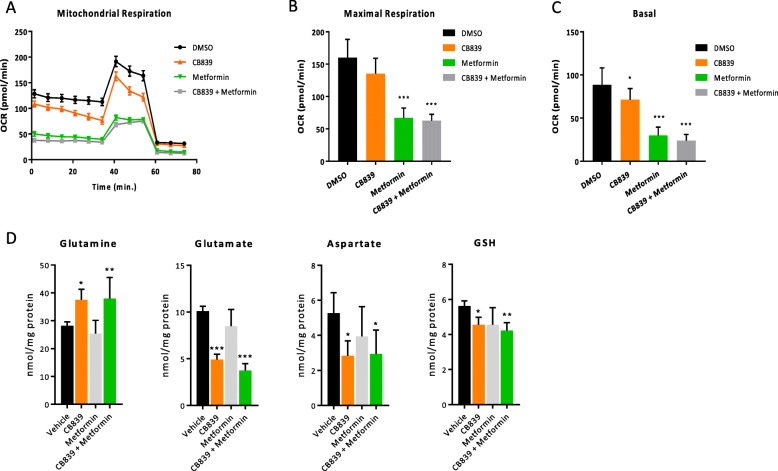
Metabolic analysis of MG63.3 cells treated with CB-839 and metformin alone and in combination in vivo. **a**–**c** Cellular oxygen consumption rates (OCR) of CB-839, metformin, and combination-treated MG63.3 cells were measured using a Seahorse Bioscience XF^e^96 Extracellular Flux Analyzer. **d** Glutamate-related metabolites quantified upon CB8-39, metformin and combo treatment in MG63.3 cells (in quadruplex) by ^1^H NMR spectroscopy. Data displayed as mean ± SD, *p < 0.05, **p < 0.01, ***p < 0.001 (ANOVA followed by multiple comparisons “vehicle vs each treatment”)

To functionally validate the findings of the cell growth inhibition by the combination treatment, we evaluated cellular metabolic changes in vitro using Seahorse extracellular flux analysis and metabolic profiling by ^1^H NMR. Cellular respiration was obtained from cells treated with CB-839, metformin, or a combination of both. Mitochondrial bioenergetic profiles (generated using the Seahorse XF Cell Mito Stress Test) showed that a significant suppression of basal and maximal mitochondrial oxygen consumption rates (OCR) was observed in metformin alone and combination-treated OS cells (Fig. 3a–c), consistent with lower TCA cycle and mithocondrial function as reported previously [30]. Metabolic profiling revealed a reduction in concentrations of glutamate, aspartate, and GSH together with increased levels of glutamine, displaying a characteristic phenotype of glutaminase inhibition [14] (Fig. 3d).

### Combination therapy with CB-839 and metformin reduces primary tumor growth in vivo

**Fig. 4:**
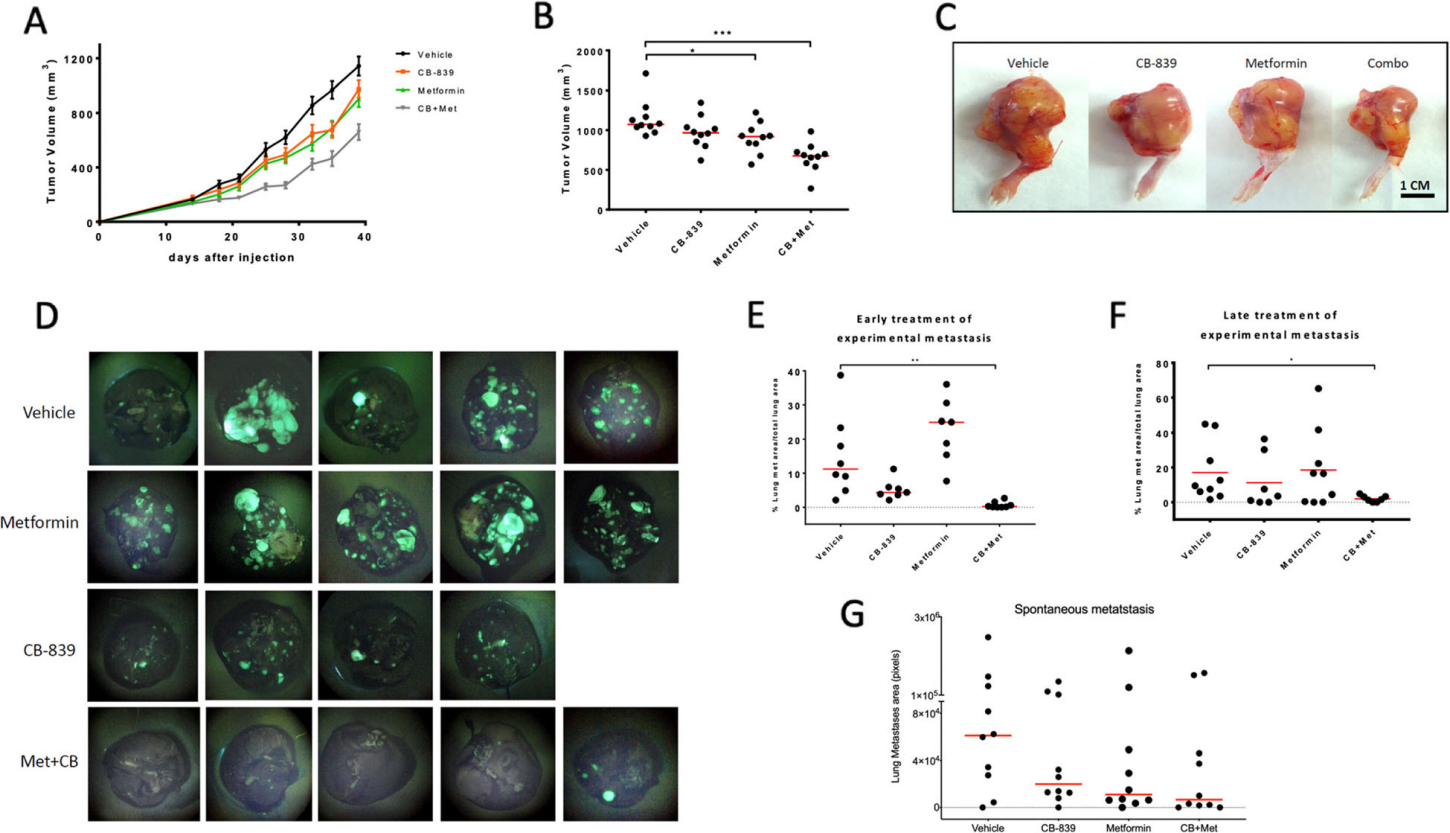
Combination therapy with CB-839 and metformin reduces OS tumor growth and inhibits metastasis in vivo. **a** Primary tumor growth rates with the treatment of vehicle control, CB-839 (200 mg/kg orally twice a day), metformin (300 mg/kg orally once a day), and in combination for 26 days (*n* ≥ 9 mice). **b** Primary tumor volumes at day 26 post initial treatment. A significant inhibition of primary tumor growth (***p < 0.001) was observed with the combination treatment. Data displayed as individual points and a red bar indicating mean value (ANOVA followed by multiple comparisons “vehicle vs each treatment”). *p < 0.05, **p < 0.01, ***p < 0.001. **c** Representative images of tumors from each treatment. **d**–**f** For the experimental metastasis model, MG63.3 cells (10^4^) were intravenously injected into SCID mice. Mice were treated as above. The treatments were started at day 2 (**d**, **e**) (early treatment) or day 9 (**f**) (late treatment) for total of 30 days. **d** Representative lung fluorescent images from each treatment revealing the significant reduction of lung metastasis from mice with the combination treatment. **e**, **f** The percent lung metastases area/total lung area from each mouse were calculated as stated in the “Methods” section. CB-839 in combination with metformin significantly inhibited MG63.3 cells lung metastasis when the treatment started as the metastatic cells were just reaching the lung (**e**, *p = 0.0019) or the micro-metastases already formed in the lung (**f**, *p = 0.0371). **g** For the spontaneous metastasis model, MG63.3 cells (10^6^) were injected into mice orthotopically. The treatments started at day 30 after tumor cell injections. Lung metastases areas from each mouse were calculated as stated in the “Methods” section. (Kruskal-Wallis followed by multiple comparisons “vehicle vs each treatment”.) *p < 0.05, **p < 0.01, ***p < 0.001

Based on our in vitro findings, we further investigated if the combination of CB-839 with metformin, or each single agent alone, could inhibit tumor growth in an orthotopic OS xenograft mouse model. As shown in Fig. 4a–c, there was a slight reduction of tumor growth with single-agent treatment; however, a significant inhibition of primary tumor growth (*p* < 0.0001) was observed with combination treatment. All treatments were well-tolerated as demonstrated by observation and weekly body weight measurements. Compared to vehicle treatment, CB-839 treatment caused no significant change in body weight, metformin caused a 5% increase, and the combination resulted in a 5% decrease in body weight (Additional file 1: Fig. S2).

### Combination therapy with CB-839 and metformin reduces osteosarcoma metastasis

As the focus of this work is the impact of metabolic targeting on metastatic progression, we next performed tumor metastasis experiments with our OS experimental and spontaneous metastasis mouse models [28] to determine therapeutic efficacy of CB-839 and metformin in this context. As shown in Fig. 4d and e, CB-839 in combination with metformin significantly inhibited lung metastasis of MG63.3/GFP cells (*p* = 0.0019). The lung metastases were also examined from H&E stained paraffin sections (Additional file 1: Fig. S3A). The assay was repeated in a highly metastatic murine OS cell line, K7M2/GFP and showed similar results (Additional file 1: Fig. S3B).

To determine if CB-839 alone or in combination could also reduce lung metastasis when micro-metastases were already established, we initiated treatment 9 days after tail vein injection of highly metastatic human OS cells (MG63.3 expressing green fluorescent protein (GFP)). In these mice, the pulmonary micro-metastases (consisting of 10–50 cell clusters) were already established before the treatment began (Additional file 1: Fig. S3C), yet the significant reduction of metastasis with the combination treatment was still observed after 30 days of continuous treatment (Fig. 4f) (*p* = 0.0371).

To confirm the observations from the experimental metastasis model, we performed spontaneous metastasis experiments with orthotopic injection of MG63.3/GFP. In this well-characterized model, following tumor development at the orthotopic site, cells spontaneously metastasize to the lungs, closely modeling human disease [25, 28]. The treatments started as the primary tumor reached approximately 12 mm in diameter. Lungs were evaluated for metastasis prior to initiation of treatment, in which we demonstrated presence of < 30 metastatic cell clusters. Consistent with experimental metastasis assays, mice treated with combination of CB-839 and metformin showed marked reduction of spontaneous metastasis compared with vehicle or single-agent-treated mice (Fig. 4g). Due to the nature of the experimental model and sample size, it did not reach statistical significance. Taken together, these data suggest a therapeutic window of opportunity exists when cells are in the mid-stages of metastasis (e.g., intravascular transit from the primary tumor or upon arrival at the secondary site of growth) which is characterized by abundant cellular stress and pervasive apoptosis [31] and may be particularly vulnerable to nutrient deprivation as they attempt to colonize the lung microenvironment as single cells or small cell clusters. Further, the in vivo data are emblematic of how metabolically targeted agents should be best evaluated in physiological contexts whenever possible [32], as the in vitro and in vivo performance may not always be congruent. Further, this data highlights the performance of such agents in the metastatic setting, wherein the unique bioenergetic challenges faced by cells are not accurately recapitulated during in vitro growth conditions.

### Tumor-level metabolite changes with combined CB-839 and metformin treatment

**Fig. 5:**
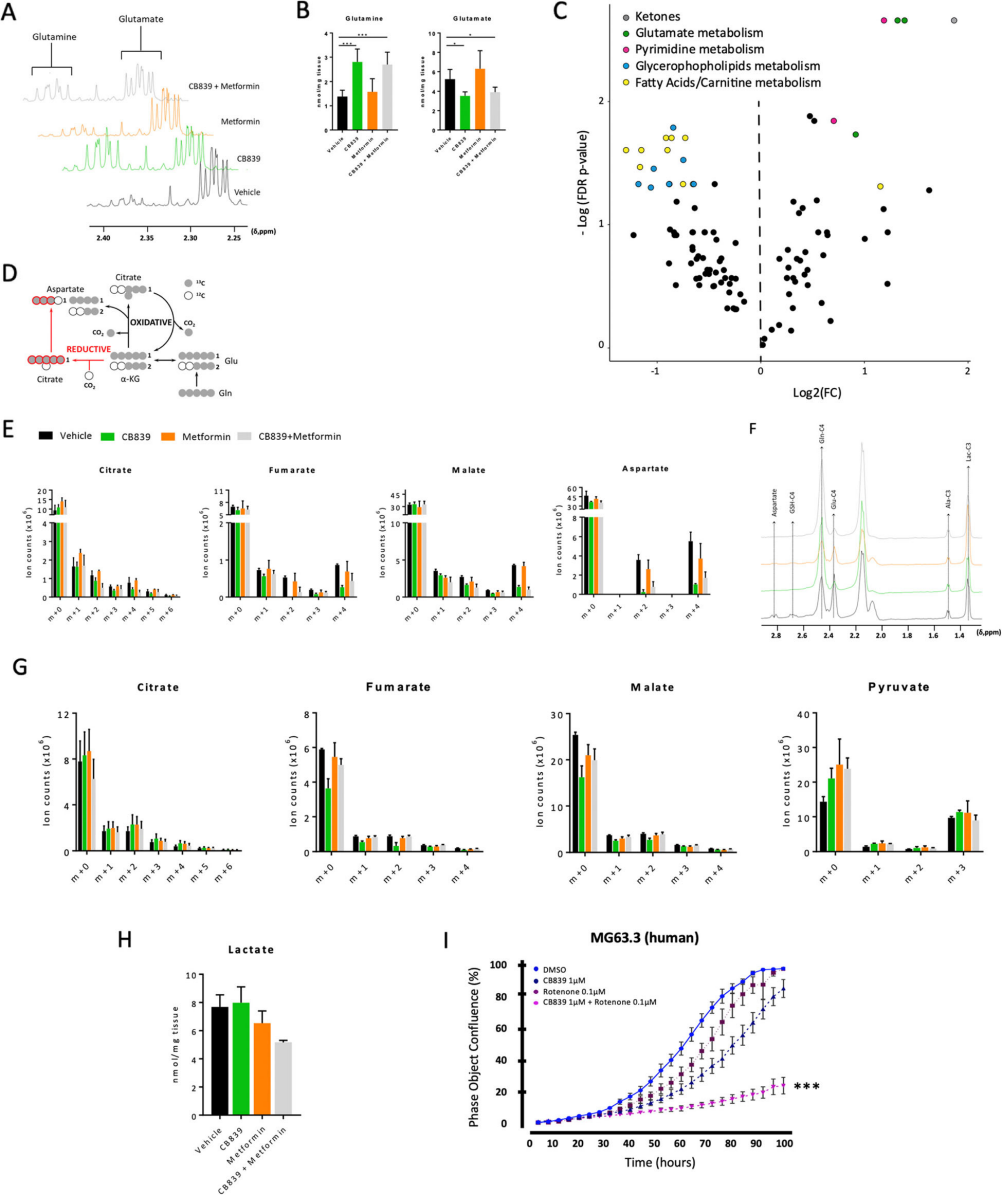
Metabolic analysis of tumors from mice treated with CB-839 and metformin alone and in combination. **a** ^1^H NMR representative spectra of tumor extracts under the 4 conditions investigated herein, displaying changes in the intensity of glutamate and glutamine resonances attributable to treatment. Intensities have been normalized to the TSP signal and to the tissue weight. Spectra displaying the multiplets arising from the protons linked to the C2 for both Glu and Gln. **b** Quantification of glutamate and glutamine from ^1^H NMR data. **c** Volcano plot displaying the main metabolites arising from the analysis vehicle vs CB-839+metformin as colored dots matching the pathway they belong to (see Additional file 1: Table S1 for the list of metabolites). **d** Roadmap of ^13^C-glutamine derived carbons throughout both, oxidative and reductive routes of TCA. **e** Tumor metabolites were analyzed by LC-MS after tail vein injections of ^13^C-Glutamine. m+0, unlabeled; m+1, m+2, m+3, m+4, m+5, and m+6 represent the degree of *m/z* increase due to ^13^C labeling. The results are presented as relative contribution of each isotopologue to the total pool at the metabolite. Data are displayed as the mean ± SD (*n* = 3–5). **f** 1d-HSQC spectrum displaying the peak intensity of protons attached to ^13^C atoms after ^13^C-glutamine injections revealing the differences arising from each treatment as the intensities of labeled metabolites. The intensities of the resonance peaks displayed were normalized to the TSP signal and to the tissue weight. **g** The same as D, but after ^13^C-glucose injection. Pyruvate enrichment analyzed by LC-MS and quantification of ^13^C-glucose-derived lactate (**h**) from NMR analysis. **i** Growth of MG63.3 cell line under CB-839, rotenone, and combination. *p < 0.05, **p < 0.01, ***p < 0.001 (ANOVA followed by multiple comparisons “vehicle vs each treatment”)

Metabolic profiling of the tissue revealed the expected changes derived from glutaminase inhibition (Fig. 5a, b). Changes in aspartate and nucleotides were also observed (Additional file 1: Fig. S4A).

Although CB-839, in combination or alone, reduced the levels of aspartate in vivo, these changes did not attain statistical significance (ANOVA followed by Sidak’s test multiple comparisons; Additional file 1: Fig S4B). Metformin treatment alone in vivo did not alter the metabolic profile of the tumors analyzed herein but did cause an increase in 3-hydroxyburate, a marker of ketosis (Additional file 1: Fig. S5B). The liquid chromatography/mass spectrometry (LC/MS) global profile of osteosarcoma tissue collected from CB-839 plus metformin treated mice revealed expected changes for glutamate pathways, specifically an increase in glutamine (FC = 2.6, FDR *p* value = 0.002) levels. Most lipids (LPC, PC, PI, PA, PS), fatty acids, glycerides, and carnitines were significantly dysregulated and presented an overall decrease upon the combination treatment (Fig. 5c). Moreover, beta-alanine (FC = 1.43, FDR *p* value = 0.014) was found to be upregulated upon this treatment. Beta-alanine serves as precursor for carnosine (FC = 2.27) and anserine (FC = 2.33) which were upregulated as a function of combination treatment. Beta-alanine is also the catabolic product of pyrimidine, which was downregulated together with uracil (Fig. 5c and Additional file 1: Table S1). These findings suggest that significant metabolites in the fatty acid and pyrimidine metabolism were ultimately downregulated in order to fuel degradation and shunt intermediates, thus driving beta-alanine and energy metabolism towards ketogenesis, as observed by the increase of 3-hydroxybutyrate levels (FC = 3.64, FDR *p* value = 0.002) in combination-treated tumor tissue.

Data derived from ^13^C-labeled glutamine and glucose were evaluated to explore the metabolic programs that osteosarcoma tumors may adopt in response to treatment with a GLS1 inhibitor alone and in combination with metformin. Bolus injection of ^13^C metabolic tracers is useful for faster turnover metabolic routes such as glycolysis and TCA but may present some limitations for slower turnover pathways such as protein and lipids synthesis [33]. We chose to focus on TCA functionality and glycolysis to avoid the confounding factors that could arise from ^13^C incorporation into lipids and proteins following our protocol.

^13^C_5_-Glutamine tracing revealed that both CB-839 and its combination with metformin reduced the levels of m+4 and m+2 TCA metabolites, which are directly derived from glutamine and follow the oxidative direction of the TCA cycle (Fig. 5d, e). We did not observe any increase in the m+3 isotopologues of malate and fumarate, neither in the levels of m+5 citrate upon any treatment, which indicates the lack of reductive carboxylation as a response to treatment. Total levels of downstream metabolites of glutamate, such as aspartate and glutathione, were also reduced after CB-839 treatment, the latter metabolites also being affected by metformin-only treatment (Fig. 5f and Additional file 1: Figure S6).

The distribution of isotopologues derived from ^13^C-labeled glucose was not significantly affected from any of the treatments investigated herein. We observed that osteosarcoma tissue displays a poor activity of pyruvate carboxylase, since the levels of the citrate isotopologue m+5 were only about 2% (compared to 15% of m+2). Pyruvate carboxylase increased activity has been reported as a potential metabolic mechanism for tumor growth in situations where GLS activity is challenged [34, 35] However, a decrease in lactate levels with metformin or combination treatment was observed via NMR. Those changes in lactate levels were accompanied by reduced glucose-derived pyruvate m+3 with combination treatment (Fig. 5g, h), indicating a global impact on glycolytic function in this context.

Metformin is known to induce multiple pharmacologic effects; however, the major effect of metformin treatment in cancer appears to be the inhibition of mitochondrial complex I [36]. To test this hypothesis, we predicted that if we substituted metformin with a mitochondrial complex I inhibitor in our combination therapy, we should see the similar effect on OS cell proliferation. As shown in Fig. 5i, replacing metformin with a specific inhibitor targeting mitochondrial complex I (rotenone) in the combination treatment with CB-839, altered the cell growth of MG63.3 OS cell line similarly to the combination of metformin and CB-839.

## Discussion

In recent years, much emphasis has been placed on drug development efforts linked to specific genetic drivers such as mutated tumor suppressor genes or oncogenes; in some cases, this approach has been very effective and successful in leading to improved outcomes for patients. However, in many tumors, and specifically in osteosarcoma, there is no known “key” driver mutation. Previous work in our lab and others indicates that cellular bioenergetics are linked to the metastatic phenotype [9, 37, 38]. To expand on this observation, we used multi-parametric metabolic profiling of treated xenografted primary tumors of highly metastatic human and murine OS cell lines, and then extrapolated the findings to help explain the significant inhibition of metastasis observed in both experimental and spontaneous models.

We demonstrate that highly metastatic OS cells rely on glutamine as a key nutrient source for cell proliferation both in vivo and in vitro. Inhibition of glutamine anaplerosis with the GLS1 inhibitor CB-839 reduces OS primary tumor growth and metastasis progression in multiple mouse models. However, the maximum reduction of cancer cell metastasis was achieved with the combination treatment of metformin and CB-839 (Fig. 4d), suggesting a critical threshold for ready access to glutamine to fuel the TCA cycle as well as other critical cellular processes exists during certain stages of tumor progression, and that this essential reliance on specific nutrients is reflective of the state of cellular stress during growth. We hypothesize that, within the OS cell lines tested, the impact of drug treatment on in vitro vs. in vivo cell proliferation is connected to the inherent stresses faced by the cells in specific growth conditions. Within conventional culture conditions, cells have ready accessed to supraphysiologic levels of various nutrients provided in culture media, thus inhibition of GLS1 only imparts a modest impact on OS cell growth. However, when cells are actively proliferating and competing for nutrients within the in vivo tumor environment, while also managing the inherent stresses associated with metastasis, interference with nutrient utilization for critical cellular processes becomes limiting.

By extension, we hypothesize that clinical translation of these findings to patients may be most successful in the minimal residual disease setting; that is, after local tumor control has been gained through surgery and inhibition of metastatic progression at distant sites becomes the main therapeutic goal.

The inhibition of metastatic progression was also observed even if treatment was initiated after micro-metastases were established in the lung (Fig. 4f, h). Because we saw a significant impact on metastasis in multiple mouse models, we hypothesize that a therapeutic window for metabolic targeting exists as the tumor cells detach and access the vasculature for transit to a secondary site; that is, cells may be particularly vulnerable to nutrient deprivation, specifically in regard to TCA cycle functionality and forced reliance on fatty acid-derived carbon, as they attempt to colonize the lung microenvironment in small numbers. As successful cells become more established in a secondary site, the combination of glutaminase inhibition with mitochondrial complex 1 inhibition, with either metformin or another complex 1-specific inhibitor, appears to be needed to deliver a significant treatment effect; as such, these established metastases behave more like primary tumors with regard to their sensitivity to the nutrient restriction imparted through CB-839 and metformin treatment. Additionally, this data lends support for further investigation of combined use of CB-839 with other mitochondrial complex 1 inhibitors such as rotenone or others that are under development based on the data presented in Fig. 5i.

Tumor-bearing mice receiving treatment with either CB-839 alone or in combination with metformin experience significant impact on a number of key, interconnected bioenergetic pathways. Among the most noteworthy is a reduction in tumor levels of aspartate. ^13^C-glutamine tracing experiments support attribution of this finding to the diminished contribution of glutamine through the oxidative direction of the TCA cycle or by direct transamination from glutamate. Aspartate levels were also reduced in the unlabeled experiments, which account for any source of aspartate (Fig. 3d and Additional file 1: Figure S4B). Aspartate is a proteinogenic amino acid, but is also a key oxidized precursor for both purines and pyrimidines. Most mammalian cells cannot acquire aspartate from their environment as it has low cell permeability and is poorly transported by cells outside of the prostate and the nervous system [39–41], and therefore must be synthesized from TCA cycle intermediates. Further, aspartate is a limiting metabolite for cancer cell proliferation when electron transport chain in the mitochondria is inhibited (as is the impact of metformin as a complex I inhibitor) or under conditions of hypoxia [12, 42]. Our data indicates that although the global impact on aspartate with CB-839 treatment did not reach statistical significance and was not impacted by single-agent metformin, we hypothesize that this somewhat modest reduction along with other metabolic changes observed in treated tumor tissue could be sufficient to impact single cells attempting to colonize the lung microenvironment under significant cellular stress.

It has been shown that carbon from available fatty acids is used exclusively to generate non-polar material, thus fatty acid oxidation does not appear to provide carbon to non-lipid biomass [11]. Subsequently, tumor cells may not be able to support de novo gluconeogenesis or protein synthesis if fatty acid-derived carbon comprises the majority of available carbon pool for cell proliferation and survival, particularly with LC/MS data suggesting that pyrimidine and fatty acid metabolic pathways are functioning catabolically in reverse (Fig. 5c, Additional file 2: Table S1), which is evidenced by the overall decrease of biologically significant intermediates and concomitant accumulation of the precursor beta-alanine. Reductions in lactate and glucose-derived pyruvate (Fig. 5g, h) further demonstrate that combination-treated cells cannot access glycolysis-derived substrates to support cell proliferation.

## Conclusions

Novel approaches are clearly needed to target the unique biology of metastasis. However, several challenges exist, including but not limited to the available model systems and structure of clinical trials to determine the anti-metastatic potential of drugs [43]. Our approach is attractive because it combines a historically well-tolerated novel therapy (CB-839) and an approved widely prescribed drug (metformin). CB-839 is a novel glutaminase inhibitor with low nanomolar potency and good oral bioavailability [14]. Collectively, this data demonstrates that CB-839, particularly when combined with metformin or another mitochrondrial complex 1 inhibitor such as rotenone, is limiting for cell proliferation primarily through forced reliance on fatty acid-derived carbon which reduces aspartate biosynthesis and induces a ketotic state. This approach could be uniquely effective for clinical translation to patients in the setting of OS metastasis, provided that tolerable metformin and CB-839 exposures can be achieved for a clinically relevant exposure period in humans.

## Supplementary information

**Additional file 1: Figure S1.** Murine OS cells K7M2 are sensitive to glutamine depletion. Cell proliferation responses to the deprivation of three carbon sources: glucose (A), glutamine (B), both glucose and glutamine (C) or pyruvate (D). **Figure S2.** Body weights of mice treated with CB-839, metformin and combination. No significant difference was found through the treatment period. **Figure S3.** A. Representative H&E stained whole lung sections of the mice treated with CB-839, metformin and the combination. B. Experimental metastasis models of K7M2 cells treated as daily gavage of vehicle, CB-839 (200 mg/kg, twice), metformin (300 mg/kg, once) or combination (*n*=4). Bar plots and SD represent the percentage of lung metastatic area over the whole lung section. C. Representative fluorescent images of the lung micro-metastases 9 days after the intravenous injection of OS MG63.3/GFP cells. **Figure S4.** A, Metabolites found to be significantly disregulted via 1H NMR upon CB-839 treatment alone. UDP-N-acetylglucosamine (GlcNac). *t*-test followed by Welch correction, *p <0.05, **p < 0.01. B, Aspartate concentration from tissue collected from mice under all the different treatments. *p*>0.05 after ANOVA test followed by Sidak’s test. **Figure S5.** A, Pathway analysis performed using the concentration of all the metabolites identified by 1H NMR. Only relevant pathways are labeled for clarity purposes. B, 3-Hydroxybutyrate concentration obtained by 1H NMR (*p* = 0.082, *t*-test followed by Welch correction). **Figure S6.** Concentrations of 13C-Gln-derived-aspartate and GSH from mice treated with metformin, CB-839 or combination.

**Additional file 2: Table S1.** Differentially regulated metabolites displayed in the volcano plot. Metabolites ID together with their FC, *p*-values, its FDRcorrected version and Log are also displayed as well as the main metabolic pathway in which they are involved.

**Additional file 3.** Supplementary Methods.

## Data Availability

The authors declare that all data supporting the findings of this study are available within the article and its supplementary information files and from the corresponding author upon reasonable request.

## Abbreviations

BPTES: Bis-2-(5-phenylacetamido-1,3,4-thiadiazol-2-yl) ethyl sulfide
DMSO: Dimethyl sulfoxide
GFP: Green fluorescent protein
GLS1: Glutaminase 1
HPBCD: 2-hydroxypropyl-beta-cyclodextrin
HBSS: Hank’s Balanced Salt Solution
LC/MS: Liquid Chromatography/Mass Spectrometry
NAD/NADH: Nicotinamide adenine dinucleotide
NMR: Nuclear magnetic resonance
OCR: Oxygen consumption rate
OS: Osteosarcoma
SRB: Sulforhodamine B
TCA: Tricarboxylic acid
