# Supplementary material for: Glutaminase-1 (GLS1) inhibition limits metastatic progression in osteosarcoma
Source: Cancer Metab. 2020 Mar 5;8:4. doi: 10.1186/s40170-020-0209-8 (PMC7057558; doi:10.1186/s40170-020-0209-8)
Supplement: Supplementary file 1 — Additional file 1: Figure S1. Murine OS cells K7M2 are sensitive to glutamine depletion. Cell proliferation responses to the deprivation of three carbon sources: glucose (A), glutamine (B), both glucose and glutamine (C) or pyruvate (D). Figure S2. Body weights of mice treated with CB-839, metformin and combination. No significant difference was found through the treatment period. Figure S3. A. Representative H&E stained whole lung sections of the mice treated with CB-839, metformin and the combination. B. Experimental metastasis models of K7M2 cells treated as daily gavage of vehicle, CB-839 (200 mg/kg, twice), metformin (300 mg/kg, once) or combination (n=4). Bar plots and SD represent the percentage of lung metastatic area over the whole lung section. C. Representative fluorescent images of the lung micro-metastases 9 days after the intravenous injection of OS MG63.3/GFP cells. Figure S4. A, Metabolites found to be significantly disregulted via 1H NMR upon CB-839 treatment alone. UDP-N-acetylglucosamine (GlcNac). t-test followed by Welch correction, *p <0.05, **p < 0.01. B, Aspartate concentration from tissue collected from mice under all the different treatments. p>0.05 after ANOVA test followed by Sidak’s test. Figure S5. A, Pathway analysis performed using the concentration of all the metabolites identified by 1H NMR. Only relevant pathways are labeled for clarity purposes. B, 3-Hydroxybutyrate concentration obtained by 1H NMR (p = 0.082, t-test followed by Welch correction). Figure S6. Concentrations of 13C-Gln-derived-aspartate and GSH from mice treated with metformin, CB-839 or combination. [file 40170_2020_209_MOESM1_ESM.pdf]

FIGURE S1

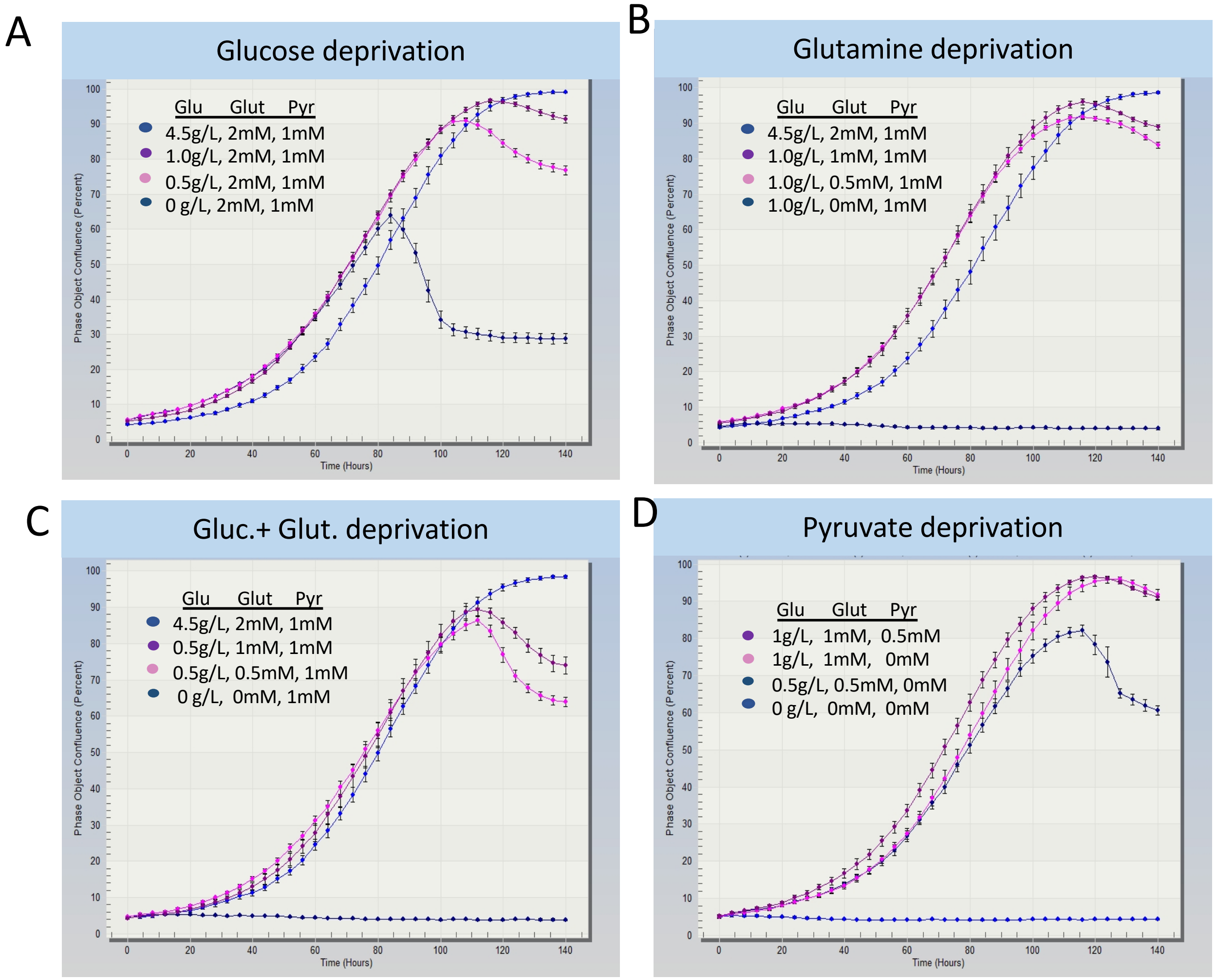

Murine OS cells K7M2 are sensitive to glutamine depletion. The proliferation responses of murine OS K7M2 cells to the deprivation of three carbon source nutrients: glucose (**A**), glutamine (**B**), both glucose and glutamine (**C**) or pyruvate (**D**) for 6 days.

FIGURE S2

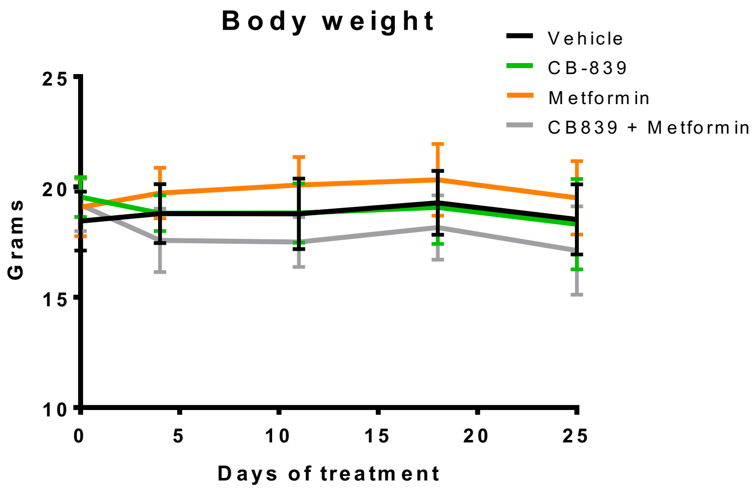

Body weights of mice treated with CB-839, metformin and combination: The measurements were taken once a week after tumor cells injection. No significant difference was found for mice body weigh through treatment period.

FIGURE S3

A

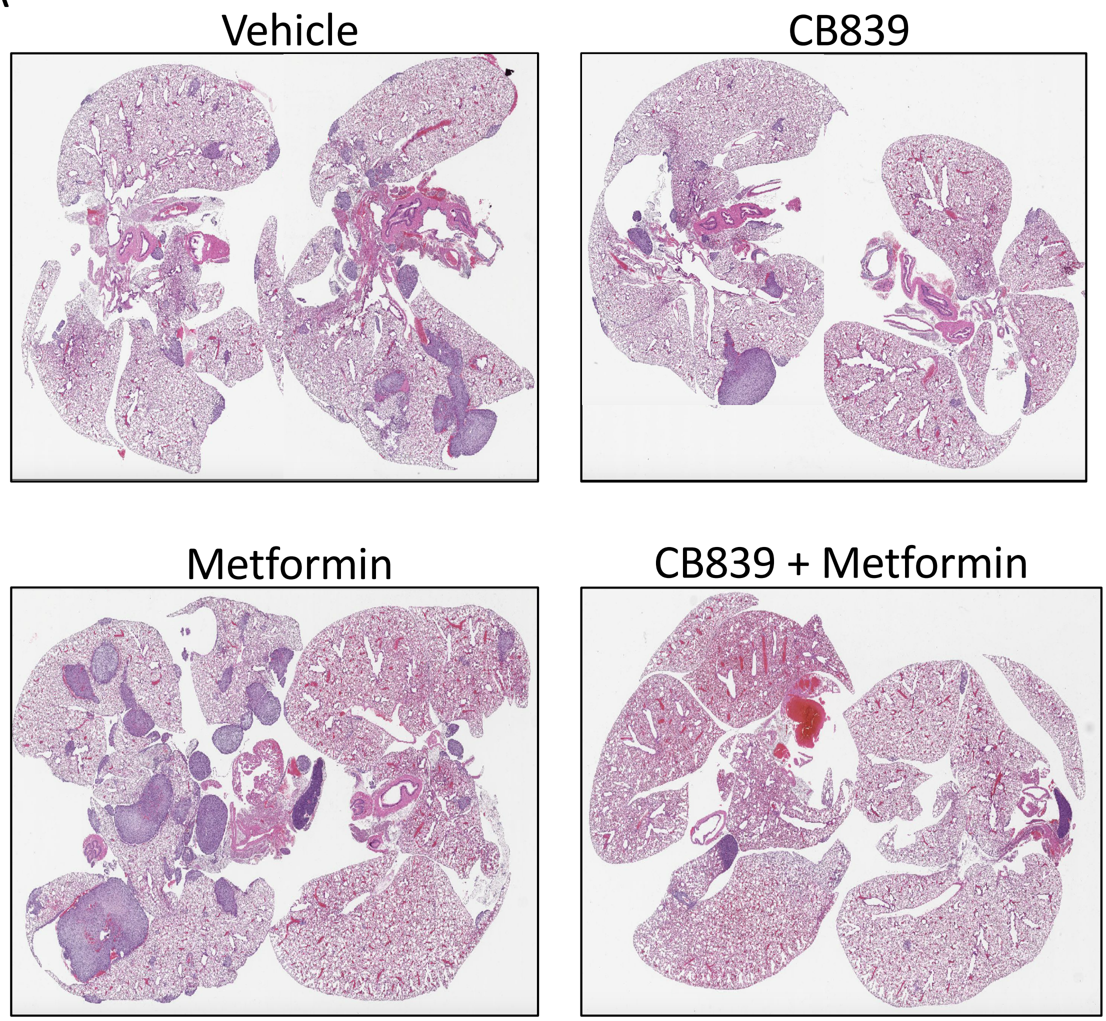
Vehicle
CB839
Metformin
CB839 + Metformin

B

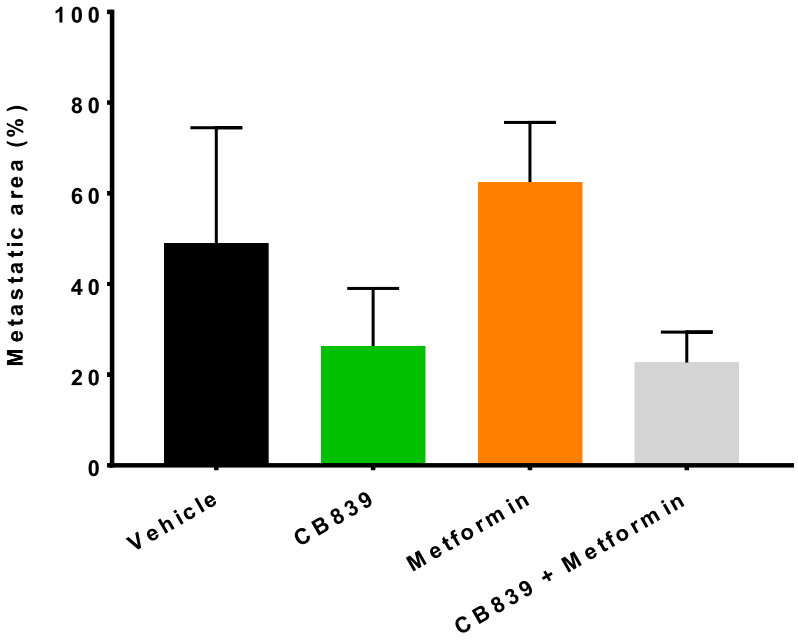
Metastatic area (%)
100
80
60
40
20
0
Vehicle
CB839
Metformin
CB839 + Metformin

C

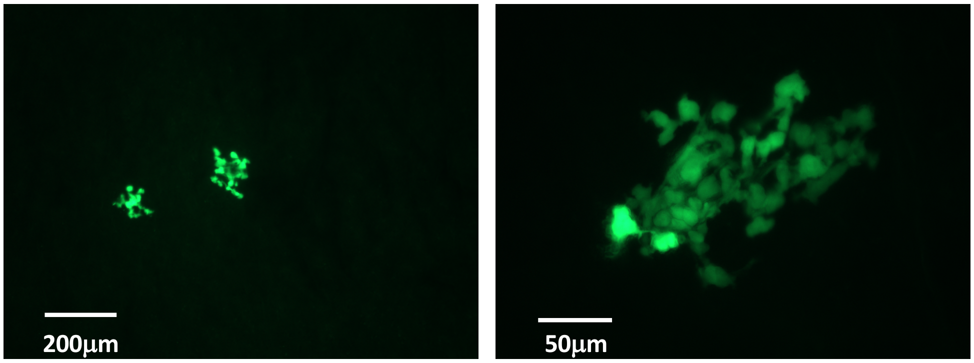

A. Representative H&E stained whole lung sections of the mice treated with CB-839, metformin and the combination. **B**. The experimental metastasis model of murine osteosarcoma K7M2 cells. Half million cells were intravenously injected into Balb/c mice. Mice received daily gavage of vehicle, CB-839 (200 mg/kg, twice a day), metformin (300 mg/kg, once a day) or combination of CB-839 and Metformin (n = 4). The treatments were started at day 2 for a total of 25 days. Data displayed as bar plots and SD representing the percentage of lung metastatic area over the whole lung section. **C**. Representative fluorescent images of the lung micro-metastases 9 days after the intravenous injection of OS MG63.3/GFP cells. Measurement as shown by the bars.

FIGURE S4

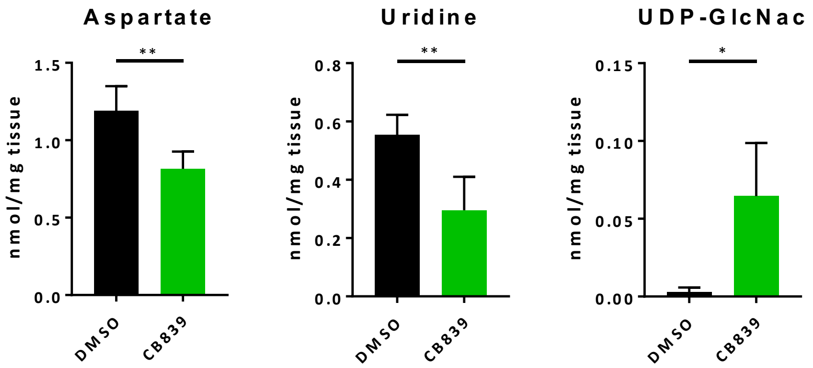

Figure S4. Metabolites found to be significantly disregulted *via* ^{1}H NMR upon CB-839 treatment alone: Aspartate, uridine and UDP-N-acetylglucosamine (GlcNac) concentrations as nmol/mg tissue. t-test followed by Welch correction, * $p < 0.05$, ** $p < 0.01$, *** $p < 0.001$.

FIGURE S5

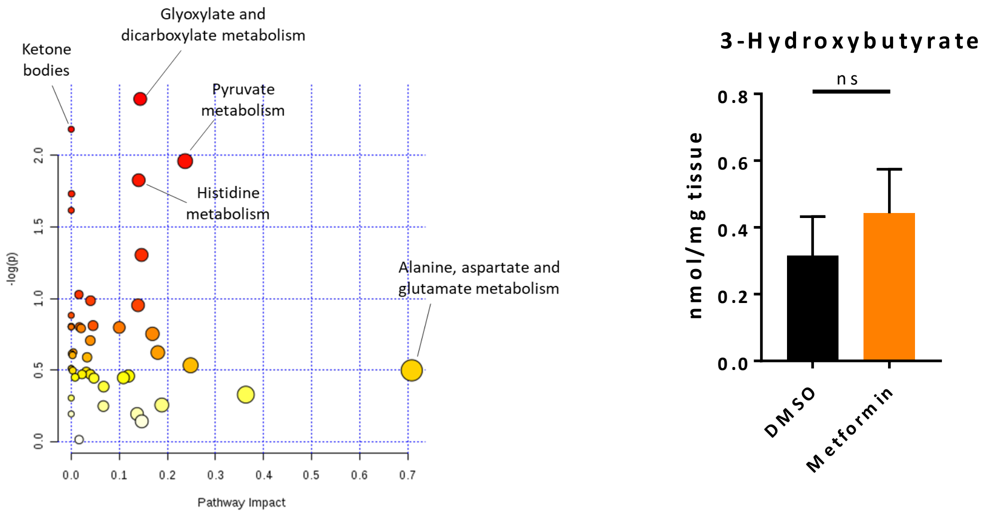

Figure S5. Pathways analysis and 3-HB levels from mice treated with metformin alone: A, Pathway analysis performed using the concentration of all the metabolites identified by ^{1}H NMR. Only relevant pathways are lebeled for clarity purposes. B, 3-Hydroxybutyrate concentration obtained by ^{1}H NMR ($p = 0.082$, t-test followed by Welch correction).

FIGURE S6

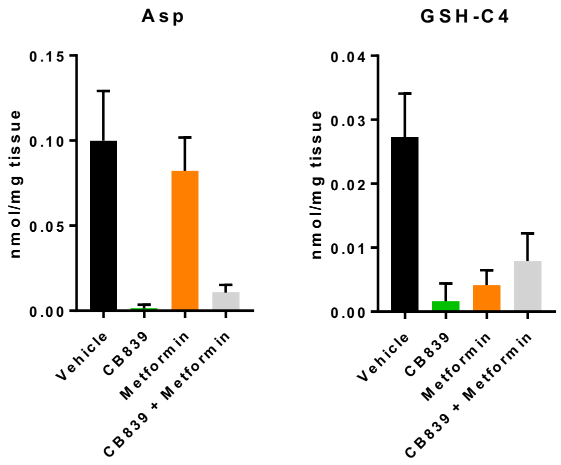

Figure S6. ^{13}C-Glutamine derived metabolites quantified by NMR: Concentrations of ^{13}C-Gln-derived-aspartate and GSH from mice treated with metformin, CB-839 or combination.
