# Supplementary material for: Glutaminase-1 (GLS1) inhibition limits metastatic progression in osteosarcoma
Source: Cancer Metab. 2020 Mar 5;8:4. doi: 10.1186/s40170-020-0209-8 (PMC7057558; doi:10.1186/s40170-020-0209-8)
Supplement: Supplementary file 3 — Additional file 3. Supplementary Methods. [file 40170_2020_209_MOESM3_ESM.pdf]

Supplementary Methods

Global metabolomic and lipidomics profiling using HPLC/MS

LC/MS global profile extraction. Osteosarcoma tissue samples were minced using surgical apparati and 450 mL of chilled MilliQ water was added. Follow-on pulverization was conducted with grinder until solution was homogenous. Samples were then snap-frozen and sonicated at 40 amps for 1 min each. Bligh and Dyer biphasic liquid extraction was performed at 2:2:1.5 chloroform/water/methanol. Chilled aqueous reagent spiked with qualitative internal standards (qIS), nitrodracylic acid and isocaramidine sulfate was added to extract solution, vortexed and incubated on ice for 10 min. The chilled chloroform reagent spiked with qIS, phenyl-N-pyridinyl acrylamide was added to the extract solution and placed on rotator for 60 min on ice. The samples were centrifuged at 12,000 rpm for 20 min at 4°C . The resulting two phases (upper hydrophilic and lower hydrophobic lipid) were separated while the remaining protein disk layer was discarded. As described by Altadill et al.[1], equivolume of acetonitrile (1:1) was added to each phase prior to centrifugation to precipitate soluble proteins. Extracts were concentrated under N_2 gas vapor, snap-frozen with dry ice and stored at -80°C .

LC/MS lipidome acquisition. The hydrophobic tissue extracts were reconstituted in reagent containing methanol:ACN:water as previously described (Altadill et al., 2017). Pooled quality control (QC) samples were composed of 10% volume from each sample. LC/MS lipidomic analysis was performed on the Agilent 6545 Quadrupole Time-of-Flight Mass Spectrometer coupled with Infinity II 1290 Liquid Chromatography Ultra-High-Pressure system. Tissue

extracts were run on an Acquity UPLC CSH 1.7 µm, 2.1 × 100 mm column (Waters Corp. Mass., USA, 186005297) using a gradient described by Altadill et al.[1].

LC/MS metabolome acquisition. Tissue hydrophilic extracts were resuspended in 60% methanol (aq) and acquired on the Agilent 6545 Qtof-MS with Infinity II 1290 UHPLC. LC/MS data acquisition was conducted through with multiple polar assays developed to achieve broad detection and high resolution of amino acids, sugar phosphates and central carbon metabolites. Global profiling of polar metabolites and relative quantification for steady state, time-dependent ^{13}C-label flux of polar metabolites was conducted on both the AdvanceBio Glycan Map 2.1 x 150 mm 2.7 µm column (Agilent Technologies, Ca., USA, 683775-913) and Acquity UPLC BEH Amide 2.1 x 100 mm, 1.7 µm column (Waters Corporation, Mass., USA, 188004801). LC/MS grade solvents and additives were used to prepare reagents, mobile phases and wash solutions, unless otherwise indicated. Wash cycles consisting of strong wash (50% Methanol, 25% Isopropanol, and 25% Water), weak wash (90% Acetonitrile and 1% Water), and seal wash (10% Isopropanol and 90% water) were utilized to eliminate carryover for consecutive injections. Glycan Map column acquisition was performed in two experiments: both positive and negative electrospray ionization (ESI) modes. Compounds were resolved over mobile phase A (10 mM ammonium acetate in 88% water and 12% acetonitrile, pH 6.85) and mobile phase B (10 mM ammonium acetate in 90% acetonitrile, pH 6.85) with column temperature 30°C at flow rate 0.3 mL/min under gradient conditions: 100% B, 0.5 min; 95% B, 2.0 min; 60% B, 3.0 min; 35% B, 5 min; hold 0.25 min; 0% B, 6 min; hold 0.5 min; 100% B, 7.25 min. BEH Amide column acquisition was performed ESI negative mode. Compounds were resolved over mobile phase A (100% water and 0.1% formic acid) and mobile phase B (100% acetonitrile and 0.1%

formic acid) with column temperature 45°C at flow rate 0.3 mL/min under gradient conditions: 99% B, 0-0.75 min; 80% B, 3.0 min; 30% B, 8.0 min; 25% B, 8.5 min; 99% B, 9.25 min. The mass analyzer parameters included drying gas temperature 250°C, sheath gas temperature 325°C, nebulizer 45 psi, skimmer 50 V, octopole radio frequency 750 V and scan rate of 5 spectra/s. In ESI positive mode experiment, ms spectra were acquired over a voltage gradient of capillary 3500 V, nozzle 2000 V, and fragmentor 165 V. In ESI negative mode experiment, mass spectra were acquired over a voltage gradient of capillary 3000 V, nozzle 2000 V, and fragmentor 100 V.

LC/MS data analysis: Prior to pre-processing each dataset, pooled QC samples (TIC, BPI and EIC) were chromatographically examined to inspect consistency of retention time and ionization levels throughout. Following acquisition, mass feature bins were defined by partitioning the m/z vs. retention time (RT) matrices into fixed width using Agilent Masshunter Profinder B.08.00. Bins were manually inspected to confirm consistent, reproducible integration for each compound of interest across all samples. Precursor m/z for each bin was determine using molecular feature extraction algorithm to deconvolute, integrate, and envelope parent ions, adducts (H-, Cl+, H+, Na+), natural isotopes and neutral losses to define each composite spectrum. Targeted ion selection and alignment parameters for logical binning of the input data were restricted to ion mass range ±5.0 mDa and retention time ± 0.4 min. Resulting neutral mass features were annotated based the in-house Personal Compound Data Library (PCDL) within mass accuracy of 0-5 mDa. Following pre-processing, the ion abundance for each sample were corrected using sample-specific weight quantification. Biostatistical analysis of LC/MS profile data was performed using Metaboanalyst 3.0 computational software. Values were corrected to sample

mass and internal standard response, then normalized to the median, and log-transformed to perform unequal variance t-test. Vista Flux software was used to conduct $^{13}C/^{15}N$ isotopologue and natural abundance comparative analysis for labelled media-substrate experiments.

[1] T. Altadill, T.M. Dowdy, K. Gill, A. Reques, S.S. Menon, C.P. Moiola, C. Lopez-Gil, E. Coll, X. Matias-Guiu, S. Cabrera, A. Garcia, J. Reventos, S.W. Byers, A. Gil-Moreno, A.K. Cheema, E. Colas, Metabolomic and Lipidomic Profiling Identifies The Role of the RNA Editing Pathway in Endometrial Carcinogenesis, Sci Rep, 7 (2017) 8803.
